# Fatty acid profile and health lipid indicies of goose meat in relation to various types of heat treatment

**DOI:** 10.1016/j.psj.2021.101237

**Published:** 2021-05-05

**Authors:** M. Wereńska, G. Haraf, J. Wołoszyn, Z. Goluch, A. Okruszek, M. Teleszko

**Affiliations:** Department of Food Technology and Nutrition, Wroclaw University of Economics and Business, 53-345 Wroclaw, Poland

**Keywords:** goose, breast muscle, heat treatment, fatty acid profile, health lipid indices

## Abstract

The effect of water bath cooking (**WBC**), oven convection roasting (**OCR**), grilling (**G**), pan-frying (**PF**) on the fatty acid profile and health lipid indices of goose meat was investigated in this study. The experimental material covered 80 breast muscles (40 with skin and subcutaneous fat and 40 without skin) cut from carcasses of 17-week-old “Polish oat geese”. The fatty acid profile of meat was determined by gas chromatography and health lipid indices were calculated. It was stated that the kind of heat treatment as well as the type of goose meat (muscles with and without skin) affected the fatty acid profile and health lipid indices. The sum of SFA was significantly higher in cooked samples for both kinds of meat than in raw ones. The cooked samples with skin had a lower increase in Ʃ SFA than the skinless meat. Boiling (meat without skin) and pan-frying (both kinds of meat) caused a slight decrease, while grilling and oven convection roasting (both kinds of meat) caused an increase of Ʃ MUFA in comparison to raw samples. Moreover, meat with skin is characterized by a higher value of Ʃ MUFA than meat without skin for all cooking methods. The Ʃ PUFA was lower in all cooked samples than in raw meat, wherein this decline was usually higher for skinned meat. The G meat was the lowest and PF the highest in Σ PUFA for both kinds of meat after heat treatment. The highest loss showed C20:4 *n-6* in OCR samples and the lowest C18:2 *n-6* in PF (both kinds of meat). Heat treatment caused an increase in the Σ PUFA *n-6/n-3* ratio, wherein the lowest value was shown by the WBC samples without skin, and the highest by OCR with skin. Water bath cooking of meat was more beneficial for consumers in terms of AI, TI, Σ DFA/Σ OFA, Σ PUFA/Σ SFA, Σ UFA/Σ SFA indexes and Σ SFA, Σ OFA values than the remaining methods.

## INTRODUCTION

The meat industry and meat processing occupy an important place in the world. Meat consumption trends vary from region to region across the world. While in some parts of the world, meat consumption might be increasing, in others, it might be decreasing, depending on the consumption trends of meat in different parts of the world (Suleman et al., 2020). For centuries, meat and its products have been essential components of our diet, providing a major proportion of consumer requirements for amino acids, fatty acids, some vitamins, and minerals. Meat is a complex food with a structured nutritional composition, which may have an effect on health. Cooked meats are an important segment of the meat industry to be used either in ready-meals, as a delicatessen product or as an ingredient in meat-based food products (Drummond and Sun, 2006; Costa et al., 2011; Grujić et al., 2014; Nowicka et al., 2018). Among many types of meat, poultry meat is particularly important. Poultry meat contains all the nutrients that meet the recommended daily allowances for humans. Therefore, in many countries, we can observe a continuous increase in the consumption of poultry (Witak, 2008; Attia et al., 2017).

Duck and goose meat, among others, is very favorable from a nutritional point of view. It contains all the essential amino acids and the highest amount of unsaturated fatty acids among all kinds of meat. Furthermore, waterfowl fat is considered to be safe for consumers due to its relatively low level of saturated fatty acids. Waterfowl production has been on an upward trend for many years and has become increasingly important around the world (Windhorst, 2011; Huang et al., 2012; Gornowicz and Lewko, 2016). In Poland, the basic breed used to produce goose meat is White Kołuda geese, and they are called “Polish oat geese,” because the birds are fattened freely with oats in the last 3 wk of rearing. Oat fattening gives unique health-promoting and taste qualities to goose meat and fat (Nowicka et al., 2018).

Consumer acceptance of meat is strongly influenced by the eating quality. Cooking methods have a great impact on the eating quality of meat (Pathare and Roskilly, 2016). The cooking operation is a procedure usually applied immediately before consumption, and meat, as well as meat-based products, are generally eaten cooked except for products that are eaten raw (Oz et al., 2016). Generally, cooking is one of the most important factors that affect the quality of meat products. Due to a series of chemical and physical reactions, cooking produces certain tenderness and taste. The cooking step is critical for destroying foodborne pathogens, assuring microbial safety and achieving meat quality (Pathare and Roskilly, 2016). During this process, meat undergoes many changes such as: weight loss, modifications of water holding capacity, texture, color, aroma development that are strongly dependent on protein denaturation, lipid and water loss.

Heat treatment can lead to undesirable changes in the nutritional value of meat, mainly due to lipid oxidation, changes in the protein fraction, and losses of some vitamins and mineral compounds (Tornberg, 2005; Walsh et al., 2010; Omojola et al., 2014; Pathare and Roskilly, 2016). Lipids are important structural and functional components of food; they have an essential effect on its quality even when their concentration is low. The oxidation products of fatty acids bound to triglycerides and phospholipids generate a series of derivatives whose biological activity is related to the initiation and progression of atherosclerosis and premature aging (Rodriguez-Estrada et al., 1997).

The effect of cooking and heating on the fatty acid composition of meat has been reported with varying results, which makes it difficult to draw general conclusions (Conchillo et al., 2004; Gerber et al., 2009; Juárez et al., 2010; Janiszewski et al., 2016). The most representative heat treatment methods used in cuisine for goose meat are: Water bath cooking, grilling, pan-frying (with and without fat or oil), deep-fat frying, oven, and microwave cooking (Oz and Celik, 2015; Oz et al., 2016). In addition, recently, the sous-vide method, especially used for beef, veal and lamb, has also been a popular heat treatment method (García-Segovia et al., 2007; Roldán et al., 2013; Roldan et al., 2015). Goose meat contains the most unsaturated fatty acids among all kinds of meat and this is very beneficial from a nutritional point of view, but on the other hand, unsaturated fatty acids can undergo oxidative processes during heat treatment.

In scientific literature, there is insufficient information on the quality of heat-treated goose meat. Therefore, the aim of this work was to describe the effect of different cooking treatments on the nutritive value of breast muscles of “Polish oat geese” by comparing: water bath cooking, oven convection roasting, grilling and pan-frying methods. In particular, the work focused on an analysis of the fatty acid profile and health lipid indices of goose meat subjected to various methods of heat treatment. Therefore, the results of this study will determine which heat treatment method used is most suitable for the meat from a nutritional point of view.

## MATERIALS AND METHODS

### Meat Samples

The experimental material was the breast (*Pectoralis major*) muscles cut from carcasses of 17-week-old “Polish oat geese”. The geese were reared in the same commercial farm and fed on the same complete concentrated diet (Wołoszyn et al., 2020b). Before the slaughter, geese were subjected to feed withdrawal for 12 h. Next, the birds were slaughtered in poultry plant, according to the regulation applied in Polish poultry industry. The eviscerated carcasses were placed in a 2°C to 4°C cooler for 24 h, and next, the breast muscles were cut out. Breast muscles were standardized for thickness and weight (average weight for breast muscles with skin and subcutaneous fat 475g ± 37g, without skin 377g ± 35g). Eighty breast muscles (40 with skin and subcutaneous fat and 40 without skin) were investigated.

### Heat Treatments

Water bath cooking, grilling, oven convection roasting and pan-frying (without fat or oil) methods were chosen. No food additives were used in the trials. A total of 16 geese breast muscles (8 samples with skin and 8 samples without skin) were used in each kind of heat treatment. The final end-point temperature for all methods was 75°C. The final cooking temperature in grilling and oven convection roasting was monitored by inserting (in the geometric center of each muscle) a Teflon-coated thermocouple (Type T, Omega Engineering Inc., Stamford, CT) connected to a temperature recorder (VAS Engineering Inc., San Diego, CA). The hand-held thermometer was used to monitor the internal temperature in boiling and pan-frying heat treatments.

### Oven Convection Roasting (OCR)

Breast muscles were wrapped in aluminum foil, then placed on trays and roasted in a forced-air convection oven (model EB7551B Fusion, Amica Ltd., Wronki, Poland) at a constant temperature (200°C). After preheating the oven to 200°C, the samples were introduced and held at this temperature for 25 min.

### Pan-Frying (PF)

The samples were placed on an electric pan (coated in Teflon, model 48155, Unold AG, Hockenheim, Germany) with a plate surface temperature of 160°C. The samples were fried and turned when the temperature in the center reached 40°C, the processing was completed after 15 min.

### Water Bath Cooking (WBC)

Each breast muscle (in a thin-walk plastic bag) was immersed in a water-bath (model SW 22, Julabo GmbH, Seelbach, Germany), at a temperature of 90°C. The bag opening was above the water surface and the overall cooking time was 30 min.

### Grilling (G)

Grilling was performed using a contact electric grill (model PD 2020R, Red Fox, Warszawa, Poland). The breast muscles were placed between 2 heating plates (the bottom and top plates were preheated to 200°C). The overall cooking time was 25 min.

After each type of heat treatment, the muscles were cooled to room temperature (for approximately 2 h). Each raw and cooked breast muscle was chopped separately (mesh diameter of 2 mm) in an electric bowl chopper (model MM/1000/887 Zelmer, Rzeszów, Poland).

### Fatty Acid Composition

The composition of fatty acids was determined using the gas chromatography technique using a Chromatograph (model 7890A, Agilent Tech., Santa Clara, CA) equipped with a flame-ionization detector (**FID**). Sample preparation, the extraction of fat, saponification, transesterification, and gas chromatography analysis of FAMEs (Fatty Acid Methyl Esters) were made in accordance with the procedure given by Wołoszyn et al. (2020a). FAMEs were identified by a comparison of the retention times with those of a mixture of external standard methyl esters from Supelco (37 FAME Mix C4-C24 Component, Sigma-Aldrich, St. Louis, MI). The fatty acids were calculated as a percentage (w/w) of total fatty acids with the Agilent ChemStation program.

### Calculation of Health Lipid Indices

The fatty acid profile was used to determine several nutritional parameters of lipids in goose breast muscles. They were calculated according to the following equations:

NVI(NutritiveValueIndex)=(C18:0+C18:1)/C16:0 (Chen et al., 2016);

AI(AtherogenicIndex)=(C12:0+4×C14:0+C16:0)/∑UFA (Ulbricht and Southgate, 1991);

TI(ThrombogenicIndex)=(C14:0+C16:0+C18:0)/[(0,5×∑MUFA)+(0,5×∑PUFAn−6)+(3×∑PUFAn−3)+(∑PUFAn−3/∑n−6)] (Ulbricht and Southgate, 1991);

OFA(C14:0+C16:0) dietary fatty acids having an undesirable hypercholesterolemic effect in humans (Janiszewski et al., 2016);

DFA(∑MUFA+∑PUFA+C18:0) dietary fatty acids having a desirable neutral hypocholesterolemic effect in humans (Janiszewski et al., 2016);

PI(PeroxidisabilityIndex)wascalculatedas:(monoenoicacid×0.025)+(dienoicacid×1)+(trienoicacid×2)+(tetraenoicacid×4)+(pentaenoicacid×6)+(hexaenoicacid×8) (Erickson, 1992).

### Statistical Analysis

The results were log-transformed to attain or approach a normal distribution, and subsequently, a two-way analysis of variance (**ANOVA**) was used in the orthogonal system. The statistical significance of the differences between the averages of the groups was verificated using Tukey's test, on the level of significance *P* ≤ 0.05, with the use of Statistica13.3 software (StatSoft Inc., 2019). The average values and their standard deviations were presented in the tables.

## RESULTS

### Fatty Acid Profile

The fatty acid profile (% of total fatty acids) in raw and cooked geese breast muscle (with and without skin) was listed in Table 1, Table 2, Table 3, while health lipid indices were listed in Table 4.

**Table 1:** Saturated fatty acid (SFA) profile of raw and cooked goose meat (% of total fatty acids) (n = 8 breast muscles with skin and n = 8 without skin for each kind of heat treatment).

Parameters	Meat	Raw meat (R)	Heat treatment					*P-value* (*P* ≤ 0.05)		
			Water bath cooking (WBC)	Grilling (G)	Oven convection roasting (OCR)	Pan frying (PF)	Total	Meat (M)	Heat treatment (T)	M x T
**C 14:0**	Without skin	^x^0.53^a^ ± 0.06	^x^0.59^a^ ± 0.06	0.46^b^ ± 0.01	0.44^b^ ± 0.03	^x^0.55^a^ ± 0.05	^x^0.51 ± 0.06	0.001	0.001	0.001
	With skin	^y^0.45 ± 0.02	^y^0.44 ± 0.07	0.44 ± 0.02	0.42 ± 0.03	^y^0.44 ± 0.04	^y^0.44 ± 0.04			
	Total	0.49^ab^ ± 0.05	0.52^a^ ± 0.10	0.45^bc^ ± 0.02	0.43^c^ ± 0.03	0.49^ab^ ± 0.07				
**C 16:0**	Without skin	^y^21.01^c^ ± 0.08	^x^20.79^d^ ± 0.02	^x^22.80^b^ ± 0.11	^x^23.04^a^ ± 0.10	^x^22.98^a^ ± 0.06	22.12 ± 1.03	0.001	0.001	0.001
	With skin	^x^21.60^c^ ± 0.03	^y^19.69^e^ ± 0.03	^y^22.66^a^ ± 0.11	^y^21.99^b^ ± 0.07	^y^20.94^d^ ± 0.03	21.38 ± 1.03			
	Total	21.31^c^ ± 0.31	20.24^d^ ± 0.58	22.73^a^ ± 0.22	22.52^ab^ ± 0.52	21.96^bc^ ± 0.76				
**C 17:0**	Without skin	^x^0.13^a^ ±0.01	0.10^bc^ ± 0.01	0.13^a^ ± 0.01	^y^0.11^b^ ± 0.01	0.09^c^ ± 0.01	0.11 ± 0.02	0.002	0.001	0.001
	With skin	^y^0.09^b^ ± 0.01	0.09^b^ ± 0.01	0.12^a^ ± 0.01	^x^0.13^a^ ± 0.01	0.08^b^ ± 0.01	0.10 ± 0.02			
	Total	0.11b ± 0.01	0.10^c^ ± 0.01	0.13^a^ ± 0.01	0.12a^b^ ± 0.01	0.09^d^ ± 0.01				
**C 18:0**	Without skin	^y^4.44^e^ ± 0.07	^y^5.92^d^ ± 0.09	^x^8.03^a^ ± 0.09	^x^7.39^b^ ± 0.08	^x^7.17^c^ ± 0.06	6.59 ± 1.29	0.001	0.001	0.001
	With skin	^x^4.70^d^ ± 0.06	^x^6.54^b^ ± 0.06	^y^7.21^a^ ± 0.09	^y^6.54^b^ ± 0.04	^y^5.82^c^ ± 0.07	6.16 ± 0.87			
	Total	4.57^d^ ± 0.15	6.23^c^ ± 0.33	7.62^a^ ± 0.44	6.97^b^ ± 0.45	6.50^bc^ ± 0.71				
**Ʃ SFA**	Without skin	^y^26.11^e^ ± 0.12	^x^27.40^d^ ± 0.10	^x^31.42^a^ ± 0.16	^x^30.98^b^ ± 0.11	^x^30.79^c^ ± 0.05	29.34 ± 2.20	0.001	0.001	0.001
	With skin	^x^26.84^d^ ± 0.08	^y^26.76^e^ ± 0.10	^y^30.43^a^ ± 0.10	^y^29.08^b^ ± 0.02	^y^27.28^c^ ± 0.09	28.08 ± 1.47			
	Total	26.47^e^ ± 0.40	27.08^d^ ± 0.34	30.93^a^ ± 0.54	30.03^b^ ± 1.00	29.04^c^ ± 1.83				

^a-e^Different letters in rows means statistically significant differences between group average, including thermal treatment (*P* ≤ 0.05).

^x-y^Different letter in columns means statistically significant differences between group average, including kind of meat (*P* ≤ 0.05).

In decreasing order of percentage, the major FAs (Fatty Acids) in the fat of raw and heat-treated (WBC, G, OCR, PF) samples, were: C18:1, C16:0, C18:2 *n-6* and C18:0. The fatty acids responded differently to heat treatment. Both, the type of goose meat (*P* = 0.001-0.008 depending on the fatty acid type) and the kind of heat treatment (*P* = 0.001-0.015 depending on the fatty acid type) affected the fatty acid profile. There was only no interaction between the type of meat × heat treatment for C20:1 (*P* = 0.264).

The sum of SFA was significantly higher in the G, OCR, PF samples than in raw meat, while for the WBC samples with skin, it was lower. Of all the heat treatment methods, meat with skin had a lower percentage (Table 1) in the sum of SFA compared to the skinless meat. This may be caused by the more cooking losses compared to meat with skin. The lowest value of Ʃ SFA was established for WBC samples (27.08%), the highest for G meat (30.93%). The higher Ʃ SFA percentage in the heat-treated meat was explained by the increase mainly of C18:0 (stearic acid). Grilling caused (for meat with and without skin) the greatest increase in the C18:0 percentage (by 53.4% and 80.9% respectively, in relation to the value for raw meat). A lower value of C18:0 was observed for PF samples with skin (5.82%) and for WBC meat without skin (5.92%) than the other ones. The WBC and OCR breast muscles with skin were characterized by the same percentage of C18:0 (6.54%). There was an increase of the C16:0 (palmitic acid) proportion for PF, G, and OCR breast muscles without skin compared to the raw meat. Only the WBC meat had a lower concentration of C16:0 than raw meat. The OCR and G total breast samples had a significantly higher percentage of C16:0 (22.52–22.73%), while WBC had a lower percentage (20.24%) than the raw meat. The C 14:0 (myristic acid), which has potential cholesterol-raising activity and therefore promotes hypercholesteremia, was detected for all cooking methods at a low proportion in the studied samples (0.44–0.59% for meat without skin and 0.42–0.44% for meat with skin) demonstrating a positive factor in their consumption. The OCR and G breast muscles without skin were characterized by a lower percentage of C14:0, whereas in the PF and WBC, it was higher than in raw meat. In the case of meat with skin, there were no significant differences in the C14:0 percentage between all heat treatment methods and raw meat, which was very beneficial from a nutritional point of view.

The Ʃ MUFA percentage in cooked meat without and with skin ranged between 46.99 and 51.16% and 51.05 and 53.26%, respectively, depending on the heat treatment methods. Significant differences were observed between the proportions of individual MUFA (Table 2). Boiling (for meat without skin) and pan-frying (for both kinds of meat) caused a slight decrease, while grilling and oven roasting (for both kinds of meat) caused an increase of Ʃ MUFA. The meat with skin is characterized by a higher value of Ʃ MUFA than meat without skin for all heat treatment methods. The percentage of the sum of MUFA was the lowest for PF samples. All types of heat treatment caused a decrease in the proportion of C16:1 in both kinds of meat. Only PF samples were characterized by a decrease in C18:1 *cis* compared to raw meat (*P* ≤ 0.05). The remaining heat treatment methods caused an increase of C18:1 *cis* in both kinds of meat (Table 2). In meat with skin, there was a significant increase in C18:1 *trans* compared to raw meat, and a higher proportion of C18:1 *trans* was produced during grilling and boiling than during pan-frying and oven convection roasting. A significant increase in C24:1 was observed for skinned meat subjected to grilling and pan-frying (Table 2).

**Table 2:** Monounsaturated fatty acid (MUFA) profile of raw and cooked goose meat (% of total fatty acids) (n = 8 breast muscles with skin and n = 8 without skin for each kind of heat treatment).

Parameters	Meat	Raw meat (R)	Heat treatment					*P-value* (*P* ≤ 0.05)		
			Water bath cooking (WBC)	Grilling (G)	Oven convection roasting (OCR)	Pan frying (PF)	Total	Meat (M)	Heat treatment (T)	M x T
**C 16:1**	Without skin	^x^4.50^a^ ± 0.09	^y^2.97^b^ ± 0.07	^y^2.71^d^ ± 0.04	^y^2.45^e^ ± 0.09	^y^2.85^c^ ± 0.07	3.10 ± 0.74	0.001	0.001	0.001
	With skin	^y^3.58^a^ ± 0.09	^x^3.42^b^ ± 0.06	^x^2.92^c^ ± 0.03	^x^2.92^c^ ± 0.03	^x^3.45^b^ ± 0.08	3.26 ± 0.29			
	Total	4.04^a^ ± 0.48	3.20^b^ ± 0.24	2.81^c^ ± 0.11	2.69^d^ ± 0.11	3.15b ± 0.32				
**C 18:1 *cis***	Without skin	^y^42.20^c^ ± 0.18	^y^42.33^c^ ±0.19	^y^45.42^b^ ± 0.23	^y^46.02^a^ ± 0.39	^y^41.04^d^ ± 0.18	^y^43.40 ± 2.04	0.001	0.001	0.001
	With skin	^x^46.48^c^ ± 0.17	^x^47.04^b^ ± 0.16	^x^47.67^a^ ± 0.21	^x^47.70^a^ ± 0.26	^x^45.14^d^ ± 0.19	^x^46.81 ± 0.98			
	Total	44.34^b^ ± 2.20	44.69^ab^ ± 2.47	46.55^a^ ± 1.18	46.86^a^ ± 0.93	43.09^b^ ± 2.14				
**C 18:1 *trans***	Without skin	^x^2.69^a^ ± 0.02	^x^2.69^a^ ± 0.07	^x^2.50^c^ ± 0.08	2.06^d^ ± 0.07	^x^2.62^b^ ± 0.06	^x^2.51 ± 0.25	0.001	0.001	0.001
	With skin	^y^1.64^c^ ± 0.03	^y^2.23^a^ ± 0.07	^y^2.25^a^ ± 0.05	2.03^b^ ± 0.08	^y^2.03^b^ ± 0.05	^y^2.04 ± 0.23			
	Total	2.17b ± 0.55	2.46^a^ ± 0.24	2.38^a^ ± 0.14	2.05^b^ ± 0.02	2.33^ab^ ± 0.31				
**C 20:1**	Without skin	0.24^b^ ± 0.03	0.26^b^ ± 0.01	0.30^a^ ± 0.03	0.26^b^ ± 0.02	0.27^ab^ ± 0.02	0.27 ± 0.03	0.001	0.015	0.264
	With skin	0.27^a^ ± 0.03	0.25^b^ ± 0.01	0.28^a^ ± 0.03	0.27^a^ ± 0.02	0.25^b^ ± 0.01	0.26 ± 0.02			
	Total	0.26^b^ ± 0.02	0.26^b^ ± 0.01	0.29^a^ ± 0.02	0.27^ab^ ± 0.02	0.26^b^ ± 0.02				
**C 24:1**	Without skin	0.17^c^ ± 0.02	^x^0.21^ab^ ± 0.01	^x^0.23^a^ ± 0.02	^x^0.19^bc^ ± 0.02	^x^0.21^ab^ ± 0.03	^x^0.20 ± 0.03	0.001	0.001	0.001
	With skin	0.17^a^ ± 0.02	^y^0.15^ab^ ± 0.01	^y^0.15^ab^ ± 0.02	^y^0.13^b^ ± 0.01	^y^0.18^a^ ± 0.02	^y^0.16 ± 0.02			
	Total	0.17^b^ ± 0.02	0.18^ab^ ± 0.04	0.19^a^ ± 0.05	0.16b ± 0.04	0.20^a^ ± 0.03				
**Ʃ MUFA**	Without skin	^y^49.80^b^ ± 0.19	^y^48.46^c^ ± 0.18	^y^51.16^a^ ± 0.20	^y^50.98^a^ ± 0.36	^y^46.99^d^ ± 0.12	^y^49.48 ± 1.62	0.001	0.001	0.001
	With skin	^x^52.13^b^ ± 0.11	^x^53.09^a^ ± 0.13	^x^53.26^a^ ± 0.21	^x^53.05^a^ ± 0.27	^x^51.05^c^ ± 0.10	^x^52.52 ± 0.87			
	Total	50.97^b^ ± 1.20	50.78^b^ ± 2.42	52.21^a^ ± 1.10	52.02^a^ ± 1.13	49.02^c^ ± 2.12				

^a-e^Different letters in rows means statistically significant differences between group average, including thermal treatment (*P* ≤ 0.05).

^x-y^Different letter in columns means statistically significant differences between group average, including kind of meat (*P* ≤ 0.05).

In our experiment, the proportion of Σ PUFA varied from 15.19% in G meat with skin to 21.00% in PF samples without skin. The sum of PUFA was significantly lower in all heat treatment samples than in raw meat, wherein this decline was in the range of 10.3 to 30.3% (depending on the heat treatment methods) for muscles without skin and in 11.8 to 24.2% for meat with skin (data calculated on the basis of Table 3). For both kinds of meat, the highest decrease compared to raw meat in Ʃ PUFA was stated for G (24.2% - meat with skin; 30.3% - meat without skin) and the lowest for PF meat (10.3% - meat without skin; 11.8% - meat with skin). The lower Ʃ PUFA percentage observed in heat-treated meat was explained by a decrease stated mainly in the proportion of individual PUFA such as: C18:2 *n-6*, C18:3 *n-3*, C20:4 *n-6*. Among these fatty acids, the highest loss was presented by C20:4 *n-6* in OCR samples without skin (60.3%) and the lowest by C18:2 *n-6* in PF muscles with skin (2.6%) (data calculated on the basis of Table 3). Losses of C18:3 *n-3* in the oven roasting method were higher than those of meat after heat treatment (both types) by other methods, too (Table 3). The lowest decline in C20:5 *n-3* was observed in the PF samples. In our experimental conditions, the changes obtained in FAs were likely due to the higher susceptibility of PUFA to oxidative degradation. For both kinds of meat and all heat treatment methods, it was observed that the size of the decrease (in relation to raw meat) in Ʃ PUFA *n-3* was higher (26.3–48.6% - data calculated on the basis of Table 3) than in Ʃ PUFA *n-6* (4.2–28.4% - data calculated on the basis of Table 3).

**Table 3:** Polyunsaturated fatty acid (PUFA) profile of raw and cooked goose meat (% of total fatty acids) (n = 8 breast muscles with skin and n = 8 without skin for each kind of heat treatment).

Parameters	Meat	Raw meat (R)	Heat treatment					*P-value* (*P* ≤ 0.05)		
			Water bath cooking (WBC)	Grilling (G)	Oven convection roasting (OCR)	Pan frying (PF)	Total	Meat (M)	Heat treatment (T)	M x T
**C 18:2 *n-6***	Without skin	^x^18.12^a^ ± 0.09	^x^16.20^c^ ± 0.09	13.28^e^ ± 0.10	^y^13.83^d^ ± 0.10	^x^17.23^b^ ± 0.11	15.73 ± 1.92	0.001	0.001	0.001
	With skin	^y^17.08^a^ ± 0.08	^y^15.90^c^ ± 0.10	13.18^e^ ± 0.06	^x^14.90^d^ ± 0.12	^y^16.62^b^ ± 0.07	15.54 ± 1.41			
	Total	17.60^a^ ± 0.55	16.05^c^ ± 0.18	13.23^e^ ± 0.10	14.37^d^ ± 0.57	16.93^b^ ± 0.33				
**C 18:3 *n-3***	Without skin	^x^1.28^a^ ± 0.08	^x^1.04^b^ ± 0.05	^x^0.71^c^ ± 0.03	^x^0.64^d^ ± 0.02	^x^0.73^c^ ± 0.04	0.88 ± 0.25	0.001	0.001	0.001
	With skin	^y^0.92^a^ ± 0.04	^y^0.77^b^ ± 0.03	^y^0.62^c^ ± 0.03	^y^0.56^d^ ± 0.02	^y^0.65^c^ ± 0.03	0.70 ± 0.13			
	Total	1.10^a^ ± 0.19	0.91^b^ ± 0.15	0.67^c^ ± 0.06	0.60^d^ ± 0.05	0.69^c^ ± 0.05				
**C 20:4 *n-6***	Without skin	^x^3.02^a^ ± 0.04	^x^1.60^d^ ± 0.04	^x^1.67^c^ ± 0.03	^x^1.20^e^ ± 0.02	^x^2.25^b^ ± 0.02	^x^1.97 ± 0.64	0.001	0.001	0.001
	With skin	^y^1.17^a^ ± 0.03	^y^1.00^b^ ± 0.03	^y^1.03^b^ ± 0.05	^y^0.82^d^ ± 0.02	^y^0.86^c^ ± 0.03	^y^0.98 ± 0.13			
	Total	2.10^a^ ± 0.68	1.30^bc^ ± 0.31	1.35^bc^ ± 0.34	1.06^c^ ± 0.20	1.56^ab^ ± 0.49				
**C 20:5 *n-3***	Without skin	^y^0.88^a^ ± 0.05	^x^0.55^c^ ± 0.03	^x^0.57^c^ ± 0.05	^x^0.54^c^ ± 0.03	^x^0.67^b^ ± 0.08	^x^0.64 ± 0.14	0.001	0.001	0.001
	With skin	^x^0.78^a^ ± 0.04	^y^0.24^c^ ± 0.04	^y^0.26^c^ ± 0.02	^y^0.27^c^ ± 0.02	^y^0.43^b^ ± 0.03	^y^0.40 ± 0.21			
	Total	0.83^a^ ± 0.07	0.40^c^ ± 0.16	0.42^c^ ± 0.16	0.41^c^ ± 0.15	0.55^b^ ± 0.14				
**C 22:6 *n-3***	Without skin	0.12^a^ ± 0.01	0.10^b^ ± 0.02	0.10^b^ ± 0.02	0.12^a^ ± 0.01	0.12^a^ ± 0.01	0.11 ± 0.02	0.008	0.002	0.007
	With skin	0.11 ± 0.01	0.09 ± 0.02	0.11 ± 0.01	0.10 ± 0.02	0.10 ± 0.02	0.10 ± 0.02			
	Total	0.12^a^ ± 0.01	0.10^b^ ± 0.02	0.11^ab^ ± 0.02	0.11^ab^ ± 0.02	0.11^ab^ ± 0.02				
**Ʃ PUFA *n-3***	Without skin	^x^2.28^a^ ± 0.08	^x^1.70^a^ ± 0.05	^x^1.38^d^ ± 0.07	^x^1.31^d^ ± 0.01	^x^1.52^c^ ± 0.10	^x^1.64 ± 0.36	0.001	0.001	0.001
	With skin	^y^1.80^a^ ± 0.05	^y^1.11^c^ ± 0.05	^y^0.99^d^ ± 0.05	^y^0.94^d^ ± 0.06	^y^1.18^b^ ± 0.04	^y^1.20 ± 0.32			
	Total	2.04^a^ ± 0.26	1.40^b^ ± 0.26	1.19^bc^ ± 0.23	1.13^c^ ± 0.23	1.35^bc^ ± 0.19				
**Ʃ PUFA *n-6***	Without skin	^x^21.14^a^ ± 0.09	^x^17.80^c^ ± 0.11	^x^14.95^e^ ± 0.07	^y^15.03^d^ ± 0.07	^x^19.48^b^ ± 0.13	17.68 ± 2.48	0.001	0.001	0.001
	With skin	^y^18.23^a^ ± 0.07	^y^15.90^c^ ± 0.07	^y^14.21^e^ ± 0.11	^x^15.72^d^ ± 0.11	^y^16.48^b^ ± 0.08	16.11 ± 1.58			
	Total	19.69^a^ ± 1.52	16.85^c^ ± 1.15	14.58^e^ ± 0.40	15.38^d^ ± 0.37	17.98^bc^ ± 1.57				
**Ʃ PUFA**	Without skin	23.42^a^ ± 0.15	19.51^c^ ± 0.10	16.32^d^ ± 0.13	16.34^d^ ± 0.11	21.00^b^ ± 0.18	^x^19.32 ± 2.79	0.001	0.001	0.001
	With skin	20.03^a^ ± 0.10	17.01^c^ ± 0.09	15.19^e^ ± 0.10	16.65^d^ ± 0.14	17.66^b^ ± 0.10	^y^17.31 ± 1.61			
	Total	21.73^a^ ± 1.77	18.26^b^ ± 1.31	15.76^d^ ± 0.60	16.50^c^ ± 0.20	19.33^ab^ ± 1.75				

^a-e^Different letters in rows means statistically significant differences between group average, including thermal treatment (*P* ≤ 0.05).

^x-y^Different letter in columns means statistically significant differences between group average, including kind of meat (*P* ≤ 0.05).

### Health Lipid Indices

Both the type of goose meat (*P* = 0.001) and the kind of heat treatment (*P* = 0.001) influenced the values of health lipid indices. There were interactions between the type of meat × heat treatment for all indices (*P* = 0.001). The Σ UFA/Σ SFA, Σ PUFA/Σ SFA and Σ PUFA *n-6/n-3* ratios are parameters used to judge the meat nutritional value and the healthiness of meat fat for human consumption. In this study, the Σ UFA/Σ SFA ratios for meat after heat treatment ranged from 2.15 to 2.62 depending on the kind of meat and heat treatment methods, and were lower than for the raw sample. The heat treatment of meat with and without skin caused a decrease in the Σ UFA/Σ SFA ratio (Table 4). There were no significant differences in the Σ UFA/Σ SFA ratios between the G and the OCR samples without skin. WBC meat was characterized by a higher value of Σ UFA/Σ SFA compared to the remaining ones. The Σ UFA/Σ SFA ratio for meat after heat treatment with skin was higher than for muscles without skin (Table 4). In our experiment, the Σ PUFA/Σ SFA ratios for both kinds of meat and for all heat treatment techniques were higher than recommended (>0.45). However, the meat after heat treatment was characterized by lower values of Σ PUFA/Σ SFA than raw meat. The Σ PUFA/Σ SFA ratios ranged from 0.50 to 0.71 depending on the kinds of meat and heat treatment methods. The most favorable Σ PUFA/Σ SFA was presented by the WBC skinned samples (0.71) and PF meat without skin (0.68). The meat with skin showed lower Σ PUFA/Σ SFA ratios than samples without skin for all heat treatment methods (except OCR) (Table 4).

**Table 4:** Nutritional quality indices of the lipids in raw and cooked goose meat (n = 8 breast muscles with skin and n = 8 without skin for each kind of heat treatment).

			Heat treatment					*P-value* (*P* ≤ 0.05)		
Parameters	Meat	Raw meat (R)	Water bath cooking (WBC)	Grilling (G)	Oven convection roasting (OCR)	Pan frying (PF)	Total	Meat (M)	Heat treatment (T)	M x T
**Ʃ UFA**	Without skin	^x^73.22^a^ ± 0.27	^y^67.96^bc^ ± 0.13	^y^67.49^cd^ ± 0.26	^y^67.32^d^ ± 0.44	^y^67.99^b^ ± 0.25	68.80 ± 2.28	0.001	0.001	0.001
	With skin	^y^72.11^a^ ± 0.16	^x^70.09^b^ ± 0.16	^x^68.44^d^ ± 0.21	^x^69.71^c^ ± 0.40	^x^68.70^d^ ± 0.04	69.81 ± 1.34			
	Total	72.67^a^ ± 0.62	69.03^b^ ± 1.12	67.96^d^ ± 0.55	68.52^c^ ± 1.31	68.35^c^ ± 0.41				
**Ʃ DFA**	Without skin	^x^77.66^a^ ± 0.29	^y^73.88^d^ ± 0.18	75.52^b^ ± 0.23	^y^74.71^c^ ± 0.37	^x^75.17^b^ ± 0.27	75.39 ± 1.31	0.001	0.001	0.001
	With skin	^y^76.81^a^ ± 0.21	^x^76.63^a^ ± 0.18	75.63^c^ ± 0.23	^x^76.24^b^ ± 0.38	^y^74.51^d^ ± 0.08	75.97 ± 0.87			
	Total	77.24^a^ ± 0.50	75.26^c^ ± 1.44	75.58^b^ ± 0.23	75.48^bc^ ± 0.88	74.84^d^ ± 0.39				
**Ʃ OFA**	Without skin	^y^21.54^c^ ± 0.08	^x^21.38^d^ ± 0.08	^x^23.26^b^ ± 0.12	^x^23.49^a^ ± 0.09	^x^23.53^a^ ± 0.06	22.64 ± 0.99	0.001	0.001	0.001
	With skin	^x^22.05^c^ ± 0.03	^y^20.13^e^ ± 0.04	^y^23.10^a^ ± 0.12	^y^22.41^b^ ± 0.07	^y^21.38^d^ ± 0.02	21.81 ± 1.03			
	Total	21.80^c^ ± 0.27	20.76^d^ ± 0.66	23.18^a^ ± 0.14	22.95^b^ ± 0.57	22.46^bc^ ± 1.12				
**Ʃ DFA/Ʃ OFA**	Without skin	^x^3.61^a^ ± 0.02	^y^3.46^b^ ± 0.02	3.25^c^ ± 0.03	^y^3.18^d^ ± 0.02	^y^3.20^d^ ± 0.02	3.34 ± 0.17	0.001	0.001	0.001
	With skin	^y^3.48^b^ ± 0.01	^x^3.81^a^ ± 0.01	3.27^d^ ± 0.02	^x^3.40^c^ ± 0.02	^x^3.49^b^ ± 0.01	3.49 ± 0.18			
	Total	3.55^a^ ± 0.09	3.64^a^ ± 0.18	3.26^b^ ± 0.05	3.29^b^ ± 0.12	3.35^b^ ± 0.15				
**Ʃ UFA/Ʃ SFA**	Without skin	^x^2.81^a^ ± 0.02	^y^2.48^b^ ± 0.01	^y^2.15^d^ ± 0.02	^y^2.17^d^ ± 0.02	^y^2.21^c^ ± 0.01	2.36 ± 0.26	0.001	0.001	0.001
	With skin	^y^2.69^a^ ± 0.01	^x^2.62^b^ ± 0.01	^x^2.25^e^ ± 0.01	^x^2.40^d^ ± 0.02	^x^2.52^c^ ± 0.01	2.50 ± 0.16			
	Total	2.75^a^ ± 0.06	2.55^b^ ± 0.07	2.20^d^ ± 0.06	2.29^c^ ± 0.12	2.37^c^ ± 0.16				
**Ʃ PUFA/Ʃ SFA**	Without skin	^x^0.90^a^ ± 0.001	^x^0.71^b^ ± 0.001	^x^0.52^e^ ± 0.001	^y^0.53^d^ ± 0.001	^x^0.68^c^ ± 0.001	0.67 ± 0.14	0.001	0.001	0.001
	With skin	^y^0.75^a^ ± 0.001	^y^0.64^c^ ± 0.001	^y^0.50^e^ ± 0.001	^x^0.57^d^ ± 0.001	^y^0.65^b^ ± 0.001	0.62 ± 0.08			
	Total	0.83^a^ ± 0.08	0.68^b^ ± 0.04	0.51^d^ ± 0.01	0.55^c^ ± 0.02	0.67^b^ ± 0.02				
**Ʃ PUFA *n-6/n-3***	Without skin	^y^9.28^d^ ± 0.28	^y^10.47^c^ ± 0.35	^y^10.89^c^ ± 0.52	^y^11.47^b^ ± 0.14	^y^12.87^a^ ± 0.89	^y^11.00 ± 1.29	0.001	0.001	0.001
	With skin	^x^10.12^c^ ± 0.25	^x^14.41^b^ ± 0.61	^x^14.44^b^ ± 0.57	^x^16.83^a^ ± 0.51	^x^14.04^b^ ± 0.46	^x^13.97 ± 2.25			
	Total	9.70^c^ ± 0.51	12.44^bc^ ± 2.11	12.67^bc^ ± 1.92	14.15^a^ ± 2.82	13.46^ab^ ± 0.91				
**NVI**	Without skin	^y^2.22^b^ ± 0.01	^y^2.32^a^ ± 0.01	^y^2.34^a^ ± 0.02	^y^2.32^a^ ± 0.01	^y^2.10^c^ ± 0.01	^y^2.26 ± 0.09	0.001	0.001	0.001
	With skin	^x^2.37^d^ ± 0.01	^x^2.72^a^ ± 0.01	^x^2.42^c^ ± 0.13	^x^2.47^b^ ± 0.02	^x^2.43^c^ ± 0.01	^x^2.48 ± 0.13			
	Total	2.30^c^ ± 0.08	2.52^a^ ± 0.21	2.38^b^ ± 0.04	2.40^b^ ± 0.08	2.27^c^ ± 0.10				
**AI**	Without skin	^y^0.29^c^ ± 0.001	^x^0.32^b^ ± 0.001	^x^0.35^a^ ± 0.001	^x^0.35^a^ ± 0.001	^x^0.35^a^ ± 0.001	0.33 ± 0.02	0.001	0.001	0.001
	With skin	^x^0.31^c^ ± 0.001	^y^0.29^d^ ± 0.001	^y^0.34^a^ ± 0.001	^y^0.32^b^ ± 0.001	^y^0.31^c^ ± 0.001	0.31 ± 0.02			
	Total	0.30^d^ ± 0.01	0.30^d^ ± 0.01	0.35^a^ ± 0.01	0.34^b^ ± 0.01	0.33^c^ ± 0.01				
**TI**	Without skin	^y^0.61^d^ ± 0.001	^x^0.71^c^ ± 0.001	^x^0.84^a^ ± 0.001	^x^0.84^a^ ± 0.001	^x^0.81^b^ ± 0.001	0.76 ± 0.09	0.001	0.001	0.001
	With skin	^x^0.66^e^ ± 0.001	^y^0.70^d^ ± 0.001	^y^0.83^a^ ± 0.001	^y^0.78^b^ ± 0.001	^y^0.73^c^ ± 0.001	0.74 ± 0.06			
	Total	0.64^e^ ± 0.01	0.70^d^ ± 0.01	0.84^a^ ± 0.01	0.81^b^ ± 0.01	0.77^c^ ± 0.01				
**PI (%)**	Without skin	^x^40.63^a^ ± 0.48	^x^30.50^c^ ± 0.24	^x^26.97^d^ ± 0.38	^x^25.54^e^ ± 0.22	^x^33.92^b^ ± 0.53	31.51 ± 5.52	0.001	0.001	0.001
	With skin	^y^30.46^a^ ± 0.20	^y^24.49^c^ ± 0.23	^y^22.63^e^ ± 0.26	^y^23.31^d^ ± 0.24	^y^25.19^b^ ± 0.25	25.22 ± 2.83			
	Total	35.55^a^ ± 5.33	27.50^b^ ± 3.15	24.80^c^ ± 2.29	24.43^c^ ± 1.18	29.56^bc^ ± 4.58				

^a-e^Different letters in rows means statistically significant differences between group average, including thermal treatment (*P* ≤ 0.05).

^x-y^Different letter in columns means statistically significant differences between group average, including kind of meat (P≤0.05).

Abbreviations: AI, atherogenic index; NVI, nutritive value index; PI, peroxidizability index; TI, thrombogenic index.

Σ DFA = Σ MUFA + Σ PUFA + C18:0; Σ OFA = C14:0 + C16:0; Σ DFA/Σ OFA - hypocholesterolemic/hypercholesterolemic index.

In the present study, the Σ PUFA *n-6/n-3* ratio in the raw breast muscles of geese was higher than the adequate value. The values of the Σ PUFA *n-6/n-3* ratios were in the range of 10.47 to 12.87 for muscles without skin and 14.04 to 16.83 for meat with skin, depending on the heat treatment method (Table 4). A lower Σ PUFA *n-6/n-3* ratio was presented by the WBC and G samples without skin in comparison to OCR and PF meat. It was a consequence of the highest percentage of total PUFA *n-3* in the case of WBC muscles and the lowest proportion of Σ PUFA *n-6* for G meat. Skinned meat was characterized by a lower value of Σ PUFA *n-6/n-3* ratios than samples with skin (Table 4). Taking into account the value of Σ PUFA *n-6/n-3* in our experiment, boiling in water and grilling were the most appropriate methods of heat treatment for meat without skin, while oven convection roasting was the most unfavorable for meat with skin. There were no significant differences in the Σ PUFA *n-6/n-3* ratios between WBC, G and PF meat with skin.

Generally, a higher value of the nutritive index (NVI) was shown by heat treatment samples with skin than those without skin (Table 4). The highest value of NVI was characteristic for WBC meat with skin (2.72). It was a consequence of a higher value of C18:1 *cis*, and the lowest proportion of C16:0 among all the investigated samples. The lowest value of NVI was observed for PF samples without skin (2.10). The meat without and with skin showed an AI index of 0.32 to 0.35 and 0.29 to 0.34, respectively, and these results are lower than recommended, which was desirable from a human health point of view (Table 4). The WBC samples (both kinds) were characterized by the lowest values of the AI index; there were no significant differences in the AI indices between G, OCR and PF meat without skin. The TI index was in the range of 0.71 to 0.84 for meat without skin and 0.70 to 0.83 for meat with skin depending on the heat treatment method used. There were no significant differences in TI values between G and OCR samples without skin. All values of TI for both types of meat regardless of the heat treatment method were higher than the adequate value of 0.5, but the lowest TI was shown for WBC for both kinds of meat. The heat treatment caused an increase in the value of TI and thus its deterioration.

In the present work, it was stated that all heat treatment methods caused a decline in the PI index for both kinds of geese breast muscles (from 40.63% - raw meat without skin, and 30.46% - with skin, to 25.54% - cooked meat without skin, and 22.63% - with skin). It means that the cooked samples had a lower protective potential for coronary artery disease than the raw ones. The cooked breast muscles with skin were characterized by a lower PI value (Table 4) than the meat without skin. Among all heat treatment methods, the oven roasting had the lowest PI value for skinless meat, and G for meat with skin. The lower PI value indicates a lower susceptibility to autooxidation of fatty acids in this meat compared to the remaining ones. On the other hand, the cooked meat without skin had a greater protective potential for coronary artery disease than heat-treated muscles with skin. The research showed that the PF muscles with skin and skinless were the most prone to autooxidation (PI = 25.19 and 33.92, respectively) in comparison to other ones.

When analyzing the proportion of the hypocholesterolemic fatty acids (**DFA**) of the investigated muscles, no significant differences have been between G and PF muscles without skin (Table 4). The sum of DFA represents 73.88 to 76.63% of total fatty acids depending on the kinds of meat and heat treatment methods. The highest percentage of Σ DFA and lowest of sum hypercholesterolemic fatty acids (Σ OFA) were shown for WBC meat with skin. The ratio between the percentage of hypocholesterolemic and hypercholesterolemic fatty acids (Σ DFA/Σ OFA indexes) indicated the effects of specific fatty acids on cholesterol metabolism and higher Σ DFA/Σ OFA values are considered more beneficial for human health. The Σ DFA/Σ OFA indexes obtained in the current study ranged from 3.18 to 3.46 for meat after heat treatment without skin and were lower than for raw meat (3.61). While in the case of samples with skin, the Σ DFA/Σ OFA index for WBC (3.81) was higher than for raw meat (3.48). The Σ DFA/Σ OFA index for both types of WBC meat was higher compared to the remaining samples.

## DISCUSSION

The effects of cooking on the meat fatty acid composition are controversial in the literature and vary among studies involving different animal species, meat cuts or products, techniques of heat treatment (Dal Bosco et al., 2001; Scheeder et al., 2001; Badiani et al., 2002, 2010; Maranesi et al., 2005; Nuernberg et al., 2006; Gerber et al., 2009; Janiszewski et al., 2016; Krempa et al., 2019). The fatty acid composition of lipids may change during meat cooking as a result of chemical reactions, such as oxidation, hydrolysis and polymerization. In general, polyunsaturated fatty acids are more susceptible to oxidation (Hernández et al., 1999). The unsaturated fatty acids are more heat-labile, and as the degree of unsaturation increases, they usually become less stable, making PUFA the most unstable (Larsen et al., 2010). Several mechanisms that occur during cooking, such as water loss and lipid oxidation, diffusion and exchange, can lead to relative changes in some FA proportion (Dal Bosco et al., 2001; Alfaia et al., 2010). Also, the neutral lipid fraction represents the storage component of the lipid, whereas the polar lipid fraction is the membrane component of the cell. The fatty acid compositions of the fractions differ greatly, with polyunsaturated fatty acids located predominately in the membrane fraction. These different distributions of fatty acids between storage and membrane fraction result in different responses with cooking (Duckett and Wagner, 1998). Due to the significant diversity of the research material, its type, size and weight, the use of different heating techniques and their parameters (time, heating temperature, heating rate, temperature in the center of the sample) discussion and comparison with literature, data are often very difficult. According to some researchers, the fatty acid profile of the meat was significantly affected by cooking, while others reported no changes. Duckett and Wagner (1998) assessed changes in the fatty acid profile of the total lipid fraction of intramuscular lipids in heat-treated beef. Similarly to our observation, they stated that cooking resulted in an increase in Ʃ SFA (especially in C18:0). It is well known that myristic and palmitic acids are among the most atherogenic agents, whereas stearic acid is thought to be neutral with respect to atherogenicity, but instead considered to be thrombogenic (Attia et al., 2017). Duckett and Wagner (1998) observed the decrease in Ʃ PUFA (C18:2, C18:3) of total lipids in lean beefsteak compared to raw meat, too. The sum of MUFA did not change after broiling. From the data they presented, cooking resulted in a deterioration of the Ʃ UFA/Ʃ SFA and Ʃ PUFA/Ʃ SFA ratios. A Σ PUFA/Σ SFA ratio above 0.45 is recommended in the human diet to prevent the development of cardiovascular disease and some other diseases, including cancer. Foods with Σ PUFA/Σ SFA ratios below 0.45 have been considered undesirable for the human diet, because of their potential to induce a cholesterol increase in the blood (Mapiye et al., 2011). Dal Bosco et al. (2001) established no differences in the Ʃ SFA, Ʃ MUFA and Ʃ PUFA percentage in raw and cooked (boiled, fried, roasted) rabbit meat. Generally cooking decreased the Ʃ PUFA *n-3* proportion and caused an increase in the Ʃ PUFA *n-6/n-3* ratio from 5.4 for raw to 10.6 for roasted, 11.9 for boiled and 12.3 for fried meat. The Σ PUFA *n-6* and Ʃ PUFA *n-3* and their ratio (Σ PUFA *n-6/n-3*) are the principal fatty acids controlling the hypocholesterolemic index. Values of the Σ PUFA *n-6/n-3* ratio below 4.0 in a diet indicate desirable quantities for cardiovascular risk prevention. The Σ PUFA *n-6/n-3* ratio ranged from 5.0 to 6.0 and can be recognized as close to recommended, suggesting that these species could be categorized as beneficial to human health consumption (Fernandes et al., 2014). In our study the heat treatment increased the Σ PUFA *n-6/n-3* ratio compared to the raw meat, which was due to a much greater decrease in the proportion of Σ PUFA *n-3* than Σ PUFA *n-6*. This was not beneficial from a nutritional point of view; however, for reasons of food safety, digestibility and sensory evaluation of geese meat, heat treatment is recommended. The highest Σ PUFA *n-6/n-3* ratio was characterized for OCR meat with skin and it was around 3-times fold the current recommendation for this value. Dal Bosco et al. (2001) stated no significant differences in the Ʃ UFA/Ʃ SFA, Ʃ PUFA/Ʃ SFA ratio and the AI indexes for raw and cooked rabbit meat. However, a decreased Ʃ PUFA *n-3* percentage in all heat treatments caused a significant increase in TI index from 1.19 in raw to 1.45 in boiled meat, but the differences between cooking methods were not significant. The AI and TI indexes indicate a potential for stimulating platelet aggregation (Ghaeni and Ghahfarokhi, 2013). Thus, the smaller the AI and TI values, the greater the protective potential for coronary artery disease. In terms of human health, the AI and TI indices, which are less than 1.0 and 0.5, respectively, in the diet, are recommended (Fernandes et al., 2014). In our study all samples characterized by the AI lower and by TI value higher than recommended. The results of Echarte et al. (2003) indicate that both microwave heating and frying in olive oil significantly changed the FAs profile of beef and chicken patties. Microwave heating caused an increase in Ʃ SFA, while frying in olive oil decrease in Ʃ SFA, which was primarily the result of changes in C16:0 and C18:0. According to these authors, the percentage of Ʃ MUFA (mainly C18:1) and Ʃ PUFA (mainly C18:2 *n-6*, C18:3 *n-3* and 22:6 *n-3*) decreased after microwave heating of beef patties. In the case of chicken patties, they observed an increase in the proportion of Ʃ MUFA after microwave heating compared to raw meat, but Ʃ PUFA did not change. The use of microwave heating did not modify the Ʃ PUFA *n-6/n-3* ratio for both products, while frying caused a significant decrease in this value for beef patties (from 10.67 to 5.37). Microwave heating decreased the Ʃ UFA/Ʃ SFA relation in beef patties, whereas no modification was stated in the chicken product. In both fried products, an increase was observed in the Ʃ UFA/ Ʃ SFA ratio. Conchillo et al. (2004) calculated the Ʃ UFA/Ʃ SFA, Ʃ PUFA/Ʃ SFA, Ʃ PUFA *n-6/n-3* ratios in order to analyze in depth the fatty acid modifications of nutritional interest occurring during cooking of chicken breast muscles (grilling in sunflower oil, roasting). Roasting did not change the Ʃ UFA/Ʃ SFA and Ʃ PUFA/Ʃ SFA ratios compared with raw meat. On the contrary, a marked increment was found for both cooking methods, with grilling (mainly due to the use of oil) making the Ʃ PUFA/Ʃ SFA ratio reach the current recommendation. The Ʃ PUFA *n-6/n-3* ratio was quite high for the analyzed samples (8.51- raw, 12.30 - roasted, 28.32 - grilled). Particularly in grilled chicken breast meat, the Σ PUFA *n-6/n-3* ratio was around 5 - times fold the current recommendation for this value (Conchillo et al., 2004). In a previous study, Maranesi et al. (2005) reported that both microwave cooking and broiling modified the concentrations of some fatty acids in the cooked lamb rib-lions slightly, but significantly compared to the uncooked samples. Some SFA increased significantly, namely C14:0 (only for microwave cooked meat), C15:0, C16:0, but the Ʃ SFA increased, however, without any statistical differences between raw, microwave cooked, and broiled meat. The Ʃ MUFA in both types of heat treatment decreased slightly, but there were no significant differences compared to raw lamb loins. However, in microwaved and broiled meat, they noted a significant increase of the concentration of C18:1 *trans* compared to the uncooked samples. The Ʃ PUFA proportion in both cooking techniques decreased, but significantly only for broiled meat. Among PUFA, the C20:4 and C18:2 decreased significantly in both methods of cooking. The Ʃ PUFA/Ʃ SFA decreased significantly in both cooking techniques but Ʃ PUFA *n-6/n-3* did not change compared to raw meat. There were no significant differences between the 2 cooking methods in the Ʃ DFA/Ʃ OFA NVI, AI, TI, PI values calculated on the basis of data given for lamb meat by Maranesi et al. (2005). The results obtained in our experiments are similar to the previous data presented by Alfaia et al. (2010), where tests were performed on cooked beef meat. In their study, significant differences were observed in the FAs profile in beef meat cooked with three different methods. The authors reported an increase of Ʃ SFA in grilled, boiled and microwaved meat in comparison to the raw samples. According to these authors, some of SFA (14:0, 16:0, 17:0, and 18:0) were significantly higher in cooked meat samples than in the uncooked meat control. They observed a significant increase in the relative proportion of Ʃ MUFA (1.9% microwaved, 2.5%-boiled, 3.4%-grilled meat), which occurred after cooking too, resulting mainly from an increase in C18:1. On the other hand, cooked beef had lower concentrations of Ʃ PUFA (5.8% microwaved, 5.9%-boiled, 7.1%-grilled meat) than raw meat, due to a significant loss of some *n-6* and *n-3* PUFA. There were no significant differences in Ʃ SFA, Ʃ MUFA, Ʃ PUFA between the cooking methods. All heat treatment techniques generated the formation of transfatty acids (**TFA**). The sum of TFA, where the main was C18:1 trans-, was slightly higher in cook meat than in raw samples, but the differences were not significant. The cooked samples were characterized by the lower value of the Ʃ UFA/Ʃ SFA, Ʃ PUFA/Ʃ SFA, Ʃ DFA/Ʃ OFA ratios compared to raw meat, but Ʃ PUFA *n-6/n-3* relations were similar to those for raw samples (in our work, the Ʃ PUFA *n-6/n-3* ratios for cooked samples were higher than for raw meat). In their study, the AI indexes (0.56–0.58) for cooked meat were close to the recommended limit and TI (1.16–1.18) (calculated based on the provided data) higher. There were no significant differences for AI and TI between the cooking methods. The presented cooking methods did not worsen the nutritive index (NVI), and our data concerning WBC, G and OCR samples were in line with those. In turn, the PI index represents the relationship between the fatty acid composition of a tissue and its susceptibility to oxidation. The PI index is used to assess the stability of PUFA included in food products and to protect them from possible oxidation processes, but the higher the PI value, the greater the protective potential for coronary artery disease (Kang et al., 2005). In our study the cooked samples had a lower protective potential (**PI**) for coronary artery disease than raw ones. These findings are in agreement with the results obtained by Alfaia et al. (2010), for beef meat subjected to various methods of heat treatment.

Nudda et al. (2013) demonstrated that the cooking process changed the concentrations of almost all FAs and FAs classes of lamb meat significantly. Microwave cooking significantly decreased the ƩSFA (mainly C14:0, C16:0) and, increased the percentage of Ʃ PUFA *n-3* and Ʃ PUFA *n-6* groups, while Ʃ MUFA was not influenced by cooking. While, the cooking improved: Ʃ PUFA *n-6/n-3*, Ʃ PUFA/Ʃ SFA, Ʃ MUFA/Ʃ SFA, AI, TI, UFA/SFA indexes, opposite as in our experience. The results obtained for “Polish oat geese” were partly in line with those found by Oz and Celik (2015) for Turkish breast geese muscles subjected to various methods of heat treatment. In our study, the sum of SFA ranged from 26.76% to 31.42% depending on the type of meat and cooking methods and was similar to values stated by Oz and Celik (2015) for geese breast meat. Comparing the same methods of heat treatments, in their studies, the Ʃ SFA percentage increased with grilling, oven cooking, pan-frying without oil, and decreased with water boiling. The increase in the relative proportion of Ʃ SFA, which occurred after cooking, mainly resulted from an increase in C17:0, C18:0, C20:0. However, a slight increase in the relative proportion of ƩMUFA was observed for boiled, grilled, pan-fried without oil and oven cooked leg meat in comparison to raw samples, but there were no significant differences between the methods. The Ʃ PUFA/Ʃ SFA increased for all cooked breast meat and Ʃ PUFA *n-6/n-3* decreased, but in our experiment, this was reversed. In their study, the Ʃ UFA/Ʃ SFA ratio increased in the case of boiled and pan-fried samples and decreased in grilled and oven cooked breast meat, while we only observed a decline in the Ʃ UFA/Ʃ SFA ratio. The data concerning the AI indexes for cooked meat were similar to their values (0.27-0.33-calculated on the basis of the given values). However, the TI (0.39–0.58) and NVI (1.61–2.09) indexes for all cooked meat were lower than our results. Our results are consistent with those obtained previously by Janiszewski et al. (2016) for boiled and roasted pork meat. They established that the proportion of Ʃ SFA in the cooked (boiled) pork *longissimus lumborum* (LL) muscle was significantly (by 1.17%) higher (there were significant changes in C14:0, C17:0 and C16), and Ʃ UFA (Ʃ MUFA+Ʃ PUFA by 1.03%) lower than in the raw meat. The proportion of the sum of MUFA and PUFA did not differ significantly between samples, while their percentage minimally decreased after boiling. Significant differences were established between the proportion of individual FAs, especially from the *n-3* (C20:5, C22:5, C22:6) and *n-6* (C20:3, C20:4, C22:4) PUFA group, which decreased in the boiled samples. The Ʃ SFA and Ʃ MUFA proportions in roasted pork *triceps brachii* (**TB**) muscle were also higher than in raw meat (by 1.51% and 1.67% respectively). There was a significant increase in C14:0, C16:0, C17:0, C18:0, C:20:0, C18:1, C20:1. The proportion of Ʃ PUFA decreased by 3.31%, which was attributed mainly to the lower percentage of *n-6* (C20:2, C20:3, C20:4, C22:4) and *n-3* (C20:5, C22:5, C22:6) PUFA group. In this experiment, the differences in the Ʃ PUFA *n-6/n-3*, Ʃ UFA/Ʃ SFA ratios and the values of Ʃ DFA, Ʃ OFA for boiled and roasted meat were not significant compared to raw meat. Also, the NVI, AI, TI indexes (calculated on the basis of the data provided by the authors) for boiled and roasted samples were similar to the raw ones, while the PI values were lower. Similarly to our findings, the NVI and AI indexes for boiled (2.24; 0.48, respectively) and roasted (2.34; 0.43, respectively) meat were lower and the TI (1.07 and 0.97, respectively) was higher than recommended. On the contrary, these authors found out that grilling did not have any significant effect on the FAs profile of fat in lamb leg. In raw and cooked meat, they stated the same proportion of individual FAs and Ʃ SFA, Ʃ MUFA and Ʃ PUFA. Krempa et al. (2019) stated that the fatty acid profile in the examined mallard duck meat was dependent on the used heat treatment technique. The authors noted that the percentage of Ʃ SFA in duck meat increased from 38.59 (roasted carcass with skin) to 40.91% (roasted skinned carcass), and in duck products (meatballs fried) from 39.40 to 43.62% (meatball boiled), and was higher in comparison to raw meat (38.12%). Changes in the proportions of C12:0, C14:0, C16:0 resulted in an increase in the Ʃ SFA value. The same dependency was observed for MUFA, where the percentage of Ʃ MUFA in duck meat increased from 33.52 (roasted skinned carcass) to 38.14% (roasted carcass with skin) and in its products (meatballs fried) from 31.96 to 33.47% (meatballs boiled), and was higher in comparison to raw meat (29.83%). They observed an increase mainly in C18:1. In turn, the relative proportion of Ʃ PUFA decreased in meat from 25.57 (roasting skinned carcass) to 23.25% (roasted carcass with skin) and in meatballs from 28.63 (fried) to 22.91% (boiled), and was lower than in raw meat (32.05%). The main fatty acids which changed the profile of PUFA were C18:3 *n-3* and C20:4 *n-6* (both declined). In our experiment, we observed similarities in all cooking methods, but furthermore, the C18:2 *n-6* declined. In the course of their study, it was shown that leaving the skin on during roasting increased the percentage of Ʃ MUFA, while decreasing the proportion of Ʃ SFA and Ʃ PUFA compared to skinned carcasses**.** We stated the same for cooked geese breast muscles with and without skin. These authors showed a lower ƩPUFA *n-6/n-3* ratio in the skinned comparison to skin - on roasted mallard duck carcass. The findings concerning the Ʃ PUFA *n-6/n-3* ratio and the Ʃ UFA/Ʃ SFA, Ʃ DFA/Ʃ OFA, NVI, AI, TI indexes for breast geese without and with skin (oven roasted) were in good agreement or close to those (calculated on basis of the fatty acid profile) for roasted mallard duck meat with and without skin. Duckett and Wagner (1998) reported great differences in the fatty acid composition between lipid fractions (neutral and polar lipids), with changes most evident in the polar lipid fraction, where PUFA are primarily located. In our study, overall, cooking reduced the proportion of C18:2 *n-6*, C18:3 *n-3*, C20:4 *n-6*. C20:5 *n-3*, C22:6 *n-3* and increased the C18:0 percentage with changes in C14:0 or C16:0, too. These changes in the percentage of the various PUFA may indicate that oxidation occurred during cooking. That is why the changes observed in the partial sums of FAs in our work are likely due to the higher susceptibility of PUFA to oxidative degradation, relative to the other FAs. Also, the authors consider that changes in the fatty acid composition that occur during cooking may be overlooked when only total lipid extracts are analyzed.

In turn, Juárez et al. (2010) suggested that thermal hydrolysis, the migration of the fatty acid from muscle to other locations, the loss of volatile fatty acids, and the deactivation of enzymes that occurred during heating, may be responsible for many of the observed changes. Meanwhile, Jiang et al. (2010) reported that changes after cooking (grilling) beef steaks were different in various forms of fat. In lean beef muscle, the percentage of Ʃ SFA increased, whereas Ʃ PUFA decreased, but in the fat forming marbling (intramuscular fat), grilling decreased the proportion of Ʃ SFA, while Ʃ MUFA and Ʃ PUFA increased.

## CONCLUSIONS

The most similar Σ PUFA *n-6/n-3* ratio to raw meat was determined for WBC and G meat without skin. All types of heat treatment for both kinds of meat caused a significant increase in the Σ PUFA *n-6/n-3* ratios compared to raw meat. These values were far from the recommendations and it was a phenomenon very unfavorable from a nutritional point of view. The oven convection roasting and pan-frying methods were the least favorable for skinless meat in terms of Σ DFA/Σ OFA, Σ UFA/Σ SFA ratios. The 4 heat treatment techniques resulted in the deterioration of TI and PI indices for both types of meat. Based on the obtained results, water bath cooking was found to be the best for consumers in terms of AI, TI, Σ DFA/Σ OFA, Σ PUFA/Σ SFA, Σ UFA/Σ SFA indexes and the OFA value. This method of heat treatment provided a lower percentage of Σ SFA than the remaining ones, too. It was difficult to clearly determine which of these methods was the least desirable in terms of preserving the nutritional value of this meat. Therefore, in the next experiment using the same types of heat technique, it could be interesting to study the changes of fatty acid profiles as well as health lipid indices in different fat forms of goose meat and in the three lipid fractions (phospholipids, free fatty acids and glycerides). We would like to determine the primary and secondary products of lipid oxidation formed during cooking, too.

## DISCLOSURES

The authors declare no conflicts of interest in publication.
